# Reconstructing Networks from Profit Sequences in Evolutionary Games via a Multiobjective Optimization Approach with Lasso Initialization

**DOI:** 10.1038/srep37771

**Published:** 2016-11-25

**Authors:** Kai Wu, Jing Liu, Shuai Wang

**Affiliations:** 1 Key Laboratory of Intelligent Perception and Image Understanding of Ministry of Education, Xidian University, Xi’an 710071, China

## Abstract

Evolutionary games (EG) model a common type of interactions in various complex, networked, natural and social systems. Given such a system with only profit sequences being available, reconstructing the interacting structure of EG networks is fundamental to understand and control its collective dynamics. Existing approaches used to handle this problem, such as the lasso, a convex optimization method, need a user-defined constant to control the tradeoff between the natural sparsity of networks and measurement error (the difference between observed data and simulated data). However, a shortcoming of these approaches is that it is not easy to determine these key parameters which can maximize the performance. In contrast to these approaches, we first model the EG network reconstruction problem as a multiobjective optimization problem (MOP), and then develop a framework which involves multiobjective evolutionary algorithm (MOEA), followed by solution selection based on knee regions, termed as MOEANet, to solve this MOP. We also design an effective initialization operator based on the lasso for MOEA. We apply the proposed method to reconstruct various types of synthetic and real-world networks, and the results show that our approach is effective to avoid the above parameter selecting problem and can reconstruct EG networks with high accuracy.

One of the outstanding problems in interdisciplinary science is to identify, predict, and control nonlinear and complex systems. Much evidence has shown that interaction patterns among dynamics elements captured by complex networks play an important role in controlling the collective dynamics^1^. However, a great challenge is that the network structure and the nodal dynamics are often unknown, instead, only the limited observed time series are available. Reconstructing complex network structure and dynamics from measureable data has become a central issue in contemporary network science and engineering^2^,^3^,^4^,^5^,^6^,^7^,^8^,^9^,^10^,^11^,^12^,^13^,^14^,^15^,^16^,^17^. Typical examples include evolutionary games networks^2^,^3^,^18^, propagation networks^4^, gene regulatory networks^6^,^13^,^14^,^15^,^16^, multiphase flow system^19^,^20^,^21^,^22^,^23^,^24^,^25^, and so on.

An important class of collective dynamics is evolutionary games (EG)^26^,^27^,^28^,^29^,^30^ in the human society. Through game theory, economists can analyze how people make choices about money; biologists can explain the origin of altruism; anthropologist can disclose the diversity of human nature; neuroscientists can reveal how individuals’ strategies influence others’ emotions and behaviors. Understanding the collective dynamics of EG is important for scientists. For the criminal gang, the police need to master the relationships between the members, namely, agent-to-agent networks. However, in the real life, it is difficult to directly access to this network, and maybe only the payoff and strategy of its members are available. Therefore, our goal is to reconstruct the agent-to-agent networks from these available information, namely, profit sequences.

Recent efforts have focused on the inverse problem of EG networks where the network reconstruction problem (NRP) is converted into a sparse signal reconstruction problem that can be solved by exploiting sparse learning algorithms, such as the lasso and compressed sensing^2^,^3^,^18^. In particular, reconstructing the whole network structure can be achieved by inferring local connections of each node individually. The problem of local structure reconstruction incorporates both the natural sparsity of complex networks and measurement error (the difference between observed data and simulated data). This problem is typically solved by using sparse learning algorithms which transform two objectives into one objective by multiplying each objective with a weighting factor and then summing up all contributions. The choice of the weighting factor has a great impact on the performance of sparse learning methods. However, a shortcoming of these penalty approaches is that it is not easy to determine this key parameter which can maximize the performance. Moreover, it is impossible to conduct the cross-validation to obtain the optimal values of this key parameter, especially when given limited data disturbed by noise and unexpected factors are not enough to split test data from them. Sometimes, there is also no gold standard to implement the cross-validation. Last but not least, playing cross-validation for the lasso is time-consuming for large-scale problems. Thus, a robust and completely data-driven approach for solving this problem remains lacking.

In this paper, we develop a multiobjective network reconstruction (MNR) framework to cope with the network reconstruction problem from profit sequences based on multiobjective evolutionary algorithm (MOEA), termed as MOEANet. To overcome the shortcoming of penalty approaches, the problem of local structure reconstruction is first modelled as a multiobjective optimization problem (MOP). One objective is to minimize the difference between the input data and the simulated data; the other is to search for sparse structure. Evolutionary algorithms (EAs)^31^,^32^,^33^ are the most popular methods for solving MOPs. Therefore, we design an improved multiobjective evolutionary algorithm, and then apply it to this MOP. All solutions in the Pareto set are optima of MOPs and represent different levels of compromise between the competing objectives. Thus, we can provide these solutions with different properties for decision makers. However, sometimes, it is necessary to determine which solution in a Pareto set (PS) is the best. Knee regions^34^,^35^,^36^, where further improvement in one objective causes a rapid degradation in other objectives, have attracted considerable interest in the study of MOPs and decision makers have been shown to prefer solutions that lie in knee regions. Therefore, an angle-based method^31^,^36^ is employed to select the eclectic Pareto solution from the Pareto front (PF) produced by EAs. Finally, the whole network can then be assembled by simply matching neighboring sets of all nodes.

To validate the performance of MOEANet, in the simulations, EG model^37^,^38^ taking place on different types of networks are used. We also present data to show that knee regions exist on the PF for this problem and that optimal solutions can be found in these knee regions. The experimental results show that MOEANet is able to effectively reconstruct EG networks and eliminate the effect of the weighting factor.

## Results

### Evolutionary Games

In an evolutionary game^26^,^27^,^28^,^29^,^30^, at any time, one agent has to choose one of strategies (*S*): cooperation (*C*) or defection (*D*), which can be expressed as *S(C*) = (1, 0)^Τ^ and *S(D*) = (0, 1)^Τ^, where Τ stands for “transpose”. The payoffs of the two agents in a game are determined by their strategies and the rewards dependent on their choices are expressed by 2 × 2 payoff matrices in agreement with the four possibilities. For example, for the prisoner’s-dilemma game (PDG)^37^, the payoff matrices are


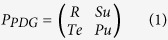


where the agents get rewards *R(Pu*) if both choose to cooperate (defect). In the remaining two cases, the defector’s and cooperator’s payoff are *Te* (temptation to defect) and *Su* (sucker’s payoff), respectively. The ranking of *Te* > *R* > *Pu* > *Su* and 2 *R* > *Te* + *Su* still holds. A spatial evolutionary PDG is introduced in ref. 37, with *R* = 1, *Pu* = *Su* = 0 and *Te* = *b*, where *b* ∈ (1, 2) is parameters characterizing the temptation to defect. In this paper, *b* is set to 1.2. At each round, all agents play game with their neighbors and gain payoffs. For agent *i*, the payoff is


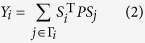


where *S*_*i*_ and *S*_*j*_ denote the strategies of agents *i* and *j* at the time and the sum is over the neighbor-connection set Γ_*i*_ of *i*. After obtaining its payoff, an agent updates its strategy according to its own and its neighbors’ payoffs, attempting to maximize its payoff at the next round.

The Fermi rule^39^ is used to simulate evolutionary-game dynamics and generate time series accordingly, which is defined as follows:


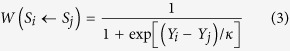


where *κ* = 0.1 characterizes the stochastic uncertainties introduced to permit irrational choices.

### Network Reconstruction from Profit Sequences in Evolutionary Games

During the evolution of EG, we assume that only the profit sequences of all agents and their strategies at each round are available. In the EG network-reconstruction problem (EGNRP), agent-to-agent interactions are learnt from profit sequences. The key to solve the EGNRP lies in the relationship between the agents’ payoffs and strategies. The interactions among agents in the network can be characterized by an *N* × *N* adjacency matrix *X* with elements *x*_*ij*_ = 1 if agents *i* and *j* are connected, and *x*_*ij*_ = 0 otherwise. Also, the interactions can be generalized straightforwardly to the weighted networks. Using the weights to characterize various interaction strengths, we define the weighted adjacency matrix *X* as: if *i* connects to *j*, *x*_*ij*_ ≥ 1; otherwise, *x*_*ij*_ = 0. The payoff of agent *i* can be expressed by


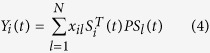


where *x*_*il*_ (*l* = 1, 2, …, *N*) represents a possible connection between agent *i* and its neighbor *l*; *x*_*il*_*S*^T^_*i*_(*t*)*PS*_*l*_(*t*) (*l* = 1, 2, …, *N*) stands for the possible payoff of agent *i* from the game with agent *i*; and *t* = 1, 2, …, *m* is the number of rounds that all agents play the game with their neighbors. The relationship among the vector *Y*_*i*_, the matrix *A*_*i*_, and the neighbor-connection vector *X*_*i*_ of agent *i* is described as follows,


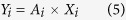


where


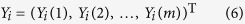



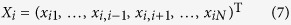



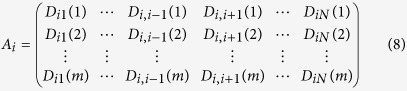


where *D*_*x,y*_(*t*) = *S*^Τ^_*x*_(*t*)*PS*_*y*_(*t*). *Y*_*i*_ can be obtained directly from the payoff data and *A*_*i*_ can be calculated from the strategy data. In a similar fashion, the neighbor-connection vectors of all other agents can be predicted, yielding the network adjacency matrix *X* = (*X*_1_, *X*_2_, …, *X*_*N*_).

Our goal is to reconstruct *X*_*i*_ from *Y*_*i*_ and *A*_*i*_. Thus, the measurement error need to be minimized. Note that the number of nonzero elements in *X*_*i*_, i.e., on average the number of real connections of node *i*, is much less than the number of all possible connections. This indicates that *X*_*i*_ is sparse, which is ensured by the natural sparsity of complex networks. There are many methods to solve this EGNRP by handling the following problem^2^,^3^,^18^.


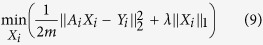


where *λ* is a constant that controls the tradeoff between the measurement error and the sparsity of networks. The *L*_1_ norm ensures the sparsity of structure, simultaneously, error control term ensures the robustness of NR against noise.

### MNR Model in Evolutionary Games

To balance the importance of measurement error with respect to the sparsity of networks, a tradeoff parameter has to be determined. A shortcoming of this method is that it introduces a parameter *λ*, and with different values of *λ*, different optimal results can be achieved. The constant is usually determined by trial and error. It is time-consuming to use the method of trial and error owing to the sizes of both the network and the data set are huge. Moreover, because of the absence of gold standards of real-world network structure, the lasso cannot use the cross-validation to obtain the optimal value of *λ*. Furthermore, when there are no enough data, we cannot split test data from raw data, especially when the performance of the method is seriously affected by the amount of data. One way of avoiding the choice of *λ* is to convert the problem into MOPs^40^,^41^,^42^. By analyzing the relationship between the Pareto optimal vectors distributed on the PF, an appropriate solution is selected from the Pareto optimal set. Considering the measurement error and the sparsity of network as two objectives, we establish the MNR model as follows,


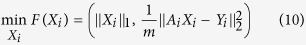


Then, we try to design a multiobjective optimization method (see Methods) to solve this model.

### Effect of the Proposed Initialization Operator

**Figure 1:**
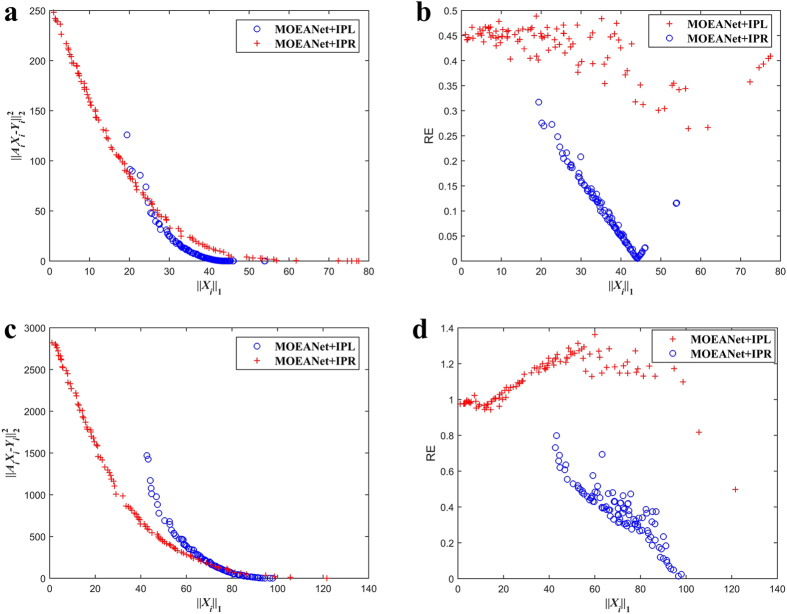
The relationship between measurement error and the sparsity of the solutions on the PF, variation of RE with change in sparsity ||*X*_*i*_||_1_. (**a**) (**b**) ER network and (**c**) (d) BA network. Different variance σ^2^ of Gaussian white noise *N*(0, σ^2^) are embedded in time series for obtaining vector *Y*_*i*_. The simulations are conducted on weighted Erdős-Rényi random networks (ER)^43^ and weighted Barabási-Albert scale-free networks (BA)^44^ with *N* = 100, σ = 0.1, the average degree 〈*k*〉 = 12, and *N*_*M*_ = 1.0, where *N*_*M*_ is the total data length *M* divided by network size *N*. Numerical simulation of EG is shown in Supplementary Note 2. In each case, the left-hand graph is a 2-D plot, graphing the relationship between the measurement error and ||*X*_*i*_||_1_. The right-hand graph shows one 2-D views of the data; variation of RE with change in sparsity ||*X*_*i*_||_1_. Each graph of Fig. 1 shows results for one sub-problem. MOEANet + IPR stands for the solutions obtained from the PF by initializing the population randomly (IPR), and MOEANet + IPL represents the solutions obtained from the PF by initializing the population with the lasso (IPL). Although space does not permit showing more examples, the graphs shown are typical of the data and usefully illustrate important observed trends. The parameters of MOEANet are showed in Supplementary Table S2.

Here, we show the effect of the proposed initialization operator on MOEANet. Figure 1(a,c) show that MOEANet + IPL can get better PF curves than MOEANet + IPR and the PS obtained by MOEANet + IPL focus on the area with small value of two objectives. Figure 1(b,d) show that MOEANet + IPL can achieve smaller value of reconstruction error (RE) (see Supplementary Note 1) than IPR. We can claim that MOEANet + IPL can effectively improve the performance of StEMO^31^. This method incorporates two additional benefits: one with greater chance to increase speed of convergence toward the PF, and another with higher probability to improve the spread of solutions along the PF.

### Existence of the Best Compromise on Knee Regions

**Figure 2:**
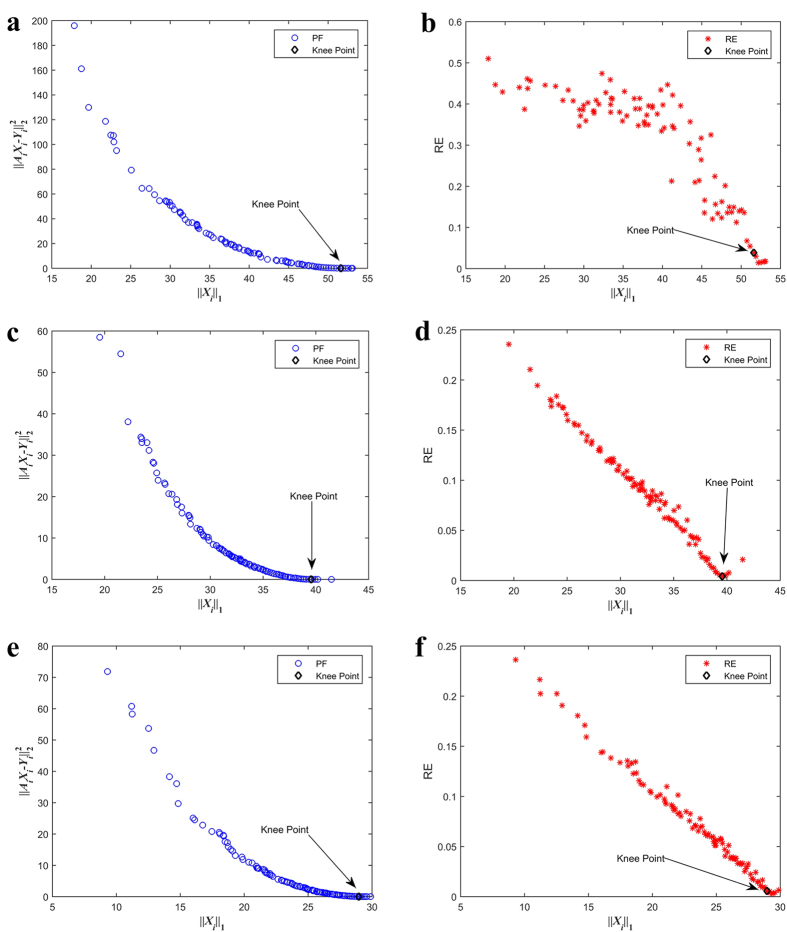
The relationships between the measurement error, RE, and the sparsity of the solutions on the PF and the position of knee point for three different values of *N*_*M*_. The simulations are conducted on ER networks with *N* = 100, σ = 0.05, and 〈*k*〉 = 12. Different variance σ^2^ of Gaussian white noise *N*(0, σ^2^) are embedded in time series for obtaining vector *Y*_*i*_. Figure 2 graphs results for three test cases where *N*_*M*_ is set to (**a**) (**b**) *N*_*M*_ = 0.4, (**c**) (**d**) *N*_*M*_ = 0.8, and (**e**) (**f**) *N*_*M*_ = 1.2. In each case, the left-hand graph is a 2-D plot, graphing the relationship between measurement error and ||*X*_*i*_||_1_. The right-hand graph shows one 2-D views of the data; variation of RE with change in sparsity ||*X*_*i*_||_1_. Each graph of Fig. 2 shows results for one example trial. Although space does not permit showing more examples of each graph for all nodes, the graphs shown are typical of the data and usefully illustrate important observed trends. The parameters of MOEANet are showed in Supplementary Table S2.

This section demonstrates that the existence of knee regions on the PF obtained from MOEA with different length of profit sequences, strength of noise, and degree of networks. For simplicity, we only consider the situation of inferring local connections of one agent and EG dynamics are simulated on weighted Erdős-Rényi random networks (ER)^43^, but the trends are similar on other types of networks. Numerical simulation of EG is shown in Supplementary Note 2. Detailed results are shown in Fig. 2, Supplementary Figs S1 and S2. As seen, although knee regions found on the PF do not give the best solution in terms of RE, the relatively optimal solution can be found.

In Fig. 2, we can observe some import properties: (1) there is an obvious knee region on the PF; (2) the found knee point provides an optimal solution, as the position of knee point is close to the point that has the smallest value of RE.

The graphs of Supplementary Fig. S1 reveal several useful trends: (1) with increasing 〈*k*〉, the position of the knee point is still close to the position that possesses the smallest RE; (2) it is evident that a knee region does exist for these problems; (3) these knee regions provide optimal solutions to these problems, because any further attempt to decrease RE will result in rapid deterioration in sparsity for only small increase in accuracy; (4) the found knee point is close to the best point having the smallest RE.

The graphs of Supplementary Fig. S2 reveal several useful trends: (1) it is evident that a knee region exists for these problems; (2) the knee point represents the best compromise between measure error and ||*X*_*i*_||_1_ which does not substantially vary with changing noise levels.

### Discussion on Parameters

**Figure 3:**
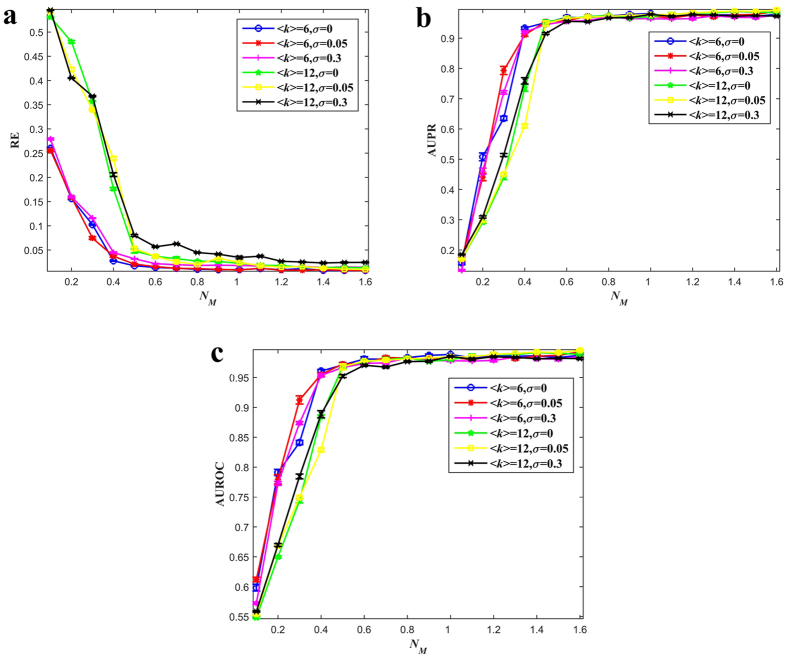
The performance of reconstructing weighted ER networks. The simulations are conducted on network size *N* = 100, 〈*k*〉 = 6 and 12, and σ = 0, 0.05, and 0.3. (**a**) RE, (**b**) AUPR, and (**c**) AUROC as functions of data amount *N*_*M*_ of time series for ER networks, respectively. Here, *N*_*M*_ is increased from 0.1 to 1.6 in steps of 0.1. Each data point is obtained by averaging over 30 independent realizations. Each solution of sub-problem is selected from the PF based on knee regions. The parameters of MOEANet are showed in Supplementary Table S2.

In this section, we study the effect of the parameters, such as data length, the average degree 〈*k*〉, and noise, on MOEANet. We simulate EG dynamics on different model-based networks (see Supplementary Note 2), including weighted Erdős-Rényi random networks (ER)^43^, weighted Barabási-Albert scale-free networks (BA)^44^, weighted Newman-Watts small-world networks (NW)^45^, and weighted Watts-Strogatz small-world networks (WS)^46^. The results are shown in Fig. 3 and Supplementary Figs S3, S4, and S5.

The results demonstrate that the length of data sequences has an important effect on the performance of MOEANet, even for small value of *N*_*M*_, most links can be identified, as reflected by the high values of the area under the receiver operating characteristic curve (AUROC) and the area under the precision-recall curve (AUPR) (see Supplementary Note 1). Still, we observe that RE decreases fast as *N*_*M*_ is increased. When *N*_*M*_ exceeds a certain value, RE is approximately 0, indicating that all link weights have been successfully predicted without failure and redundancy, despite that the link weights are random. We also examine ER networks and WS networks and observe that, to achieve the same level of accuracy, the requirement for data can be somewhat relaxed as compared with BA networks and NW networks. In the absence of noise or for small noise variance, say, σ = 0 or 0.05, high reconstruction rate can be assured by small amounts of data relative to the network size *N*. For large noise variance, say, σ = 0.3, high reconstruction rate can still be achieved based on relatively large amounts of data for different networks, manifesting the strong robustness of our method against noise in time series. We also discuss the effect of the average node degree 〈*k*〉 on MOEANet. The results demonstrate that for large value of 〈*k*〉, our method can guarantee complete identification of all links and weights. With increasing 〈*k*〉, high reconstruction rate can still be achieved needing greater *N*_*M*_ than that of sparse networks.

### Comparison of MOEANet Against the Lasso

**Figure 4:**
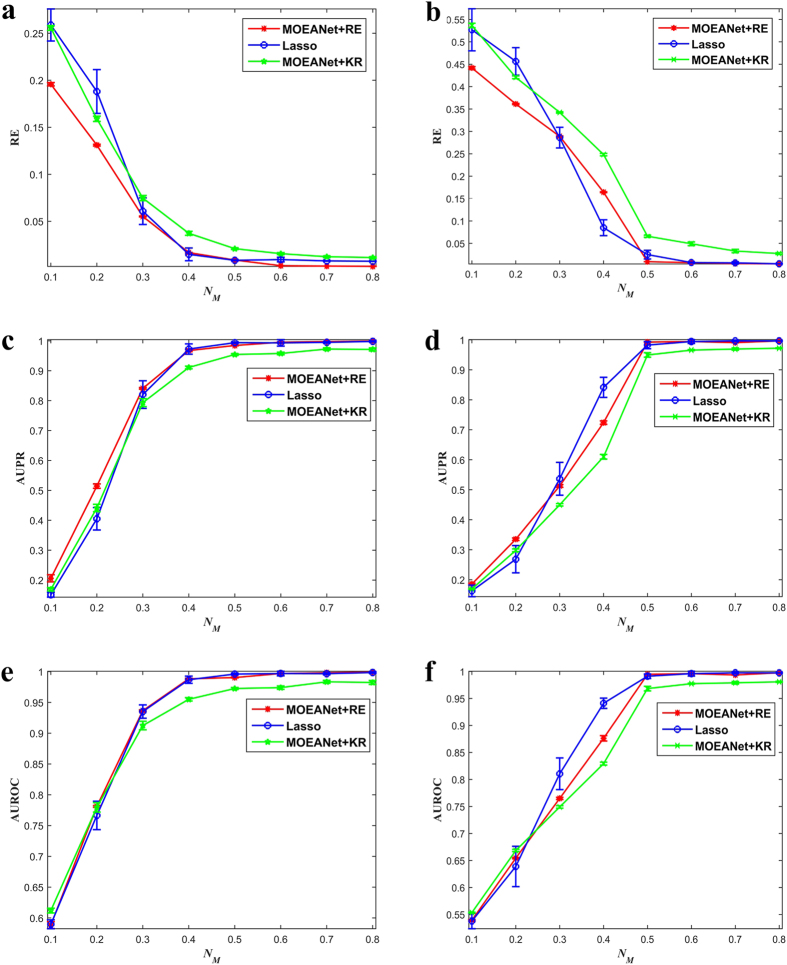
The comparison of MOEANet against the lasso. (**a**) RE, (**c**) AUPR, and (**e**) AUROC as functions of the relative data length *N*_*M*_ of time series for weighted ER networks with 〈*k*〉 = 6. (**b**) RE, (**d**) AUPR, and (**f**) AUROC as functions of the relative data length *N*_*M*_ of time series for weighted ER networks with 〈*k*〉 = 12. Here, we set *N* = 100, σ = 0.05. *N*_*M*_ is increased from 0.1 to 0.8 in steps of 0.1. For MOEANet + RE, each solution of sub-problem selected from the PF has the best *generalization ability*, namely, the smallest value of RE. For MOEANet + KR, each solution of sub-problem is selected from the PF based on knee regions. For the lasso, we set λ = 0.001, which is best value for EG network reconstruction. Each data point is obtained by averaging over 30 independent realizations. The parameters of MOEANet are showed in Supplementary Table S2.

The simulations are conducted on EG dynamic with weighted ER networks, BA networks, WS networks, and NW networks (see Fig. 4, Supplementary Figs S6, S7, and S8). As seen, AUPR and AUROC increase and RE decreases for all methods as *N*_*M*_ gets greater. Lasso works well on these cases and it obtained even less average RE and higher average AUPR and AUROC than MOEANet + KR. However, MOEANet + RE outperforms the lasso, demonstrating MOEANet can achieve better solution than the lasso, but just the angle-based method cannot find it. Furthermore, the experimental results show that although MOEANet + KR cannot find the best solution from the PF, it can obtain the relatively optimal solution. Thus, compared with the lasso, our MNR model can effectively eliminate the effect of λ in the lasso.

### Simulations on Real Networks

In this section, we test our method on eight real networks (see Table 1 and Supplementary Table S1). As seen, in terms of AUPR and AUROC, MOEANet can reconstruct EG network played on real networks with high accuracy. Precision of MOEANet can achieve approximately 1 with different real networks and the false positive rate (FPR) of MOEANet can achieve approximately 0 with different real networks which demonstrate MOEANet can fully identify null-interactions. In terms of RE, MOEANet can accurately learn the weight between agents. However, in terms of true positive rate (TPR), our approach cannot identify all agent-to-agent interactions. For large-scale real networks, namely, netscience network, MOEANet still can identify most of the interactions. For dense networks, MOEANet also can achieve high accuracy.

## Discussion

In this paper, we have developed a MNR framework to reconstruct EG networks from profit sequences. It is noteworthy that the proposed approach is quite flexible and not limited to the networked systems discussed here, such as gene regulatory networks, transportation networks, and communications networks. The contributions of this paper are summarized as follows,

1. We first model network reconstruction problems in EG network as an MOP. This way eliminates the tradeoff parameter that determines the tradeoff between reconstruction error and the sparsity of network. Our simulations also demonstrate the MNR model is efficient in EG reconstruction problem.
2. Based on the proposed MNR framework, an improved MOEA algorithm referred to as MOEANet is proposed to solve MOP. A new initial operator based on the lasso is proposed to improve the performance of MOEA, guiding the search in initialization process, and the results show the effectiveness of our improvement in initializing the population.
3. The simulations on EG dynamics simulated on weighted ER networks, BA networks, NW networks, and WS networks with various average degree and scales demonstrate that MOEANet can effectively eliminate the effect of weight factor.

In the simulations, we find that the lasso outperforms MOEANet + KR in some cases. Two factors lead to this phenomenon. On one hand, this is due to the lasso benefits from being given optimal parameter values in this simulation. However, in many situations, for example, enough data are not available, it is usually not possible to know the optimal choices of the parameter of λ in the lasso. On the other hand, it is not easy to find the exact PF and this can sometimes have an impact on how accurately we can find the knee regions. The reason that MOEANet + KR does not outperform the lasso on these cases may be that the method for detecting knee points was misled by the inclusion of a few suboptimal solutions on the estimated PF. There are many factors to affect the performance of finding knee regions. First, the problem is NP-hard, and it is hard to ensure whether the estimated PF produced by the algorithm converges to the true PF. Second, the ranges of measurement error and ||*X*_*i*_||_1_ usually have greatly different magnitudes. Finally, owing to the fixed population size, it is not easy to obtain a set of solutions that adequately sample the full range of the PF. Note that, in the worst case, even if a knee region solution in a particular problem does not turn out to provide the best solution, then the solution will still be a Pareto solution, which means that these solutions are still optimal in the sense of MOP.

To solve this problem, priori information can be employed to find optimal solution. For example, there are many Pareto solutions being in small value of the *L*_1_ norm term and big value of the error control term. In fact, full reconstruction appears when the error control term achieves approximately 0. To achieve accurate estimates of knee regions, first, we eliminate the PS with relatively big value of the error control term from the PF. A simply way is to remove top ten PS with big value of the error control term.

Eliminating indirect interactions is an important issue to solve. With a relatively small amount of data, namely, complex networks cannot be fully reconstructed, it is a genuine need to eliminate the effect of indirect interactions. However, the final solution is far from being consummated even if many works have been proposed^15^,^16^,^18^. We expect to solve this problem in future work.

In summary, we establish a diagram to reconstruct complex networks which has effectively avoided the difficulties of conventional numerical optimization methods and achieved good performance of our diagram provides an avenue on a wide range of applications in real life.

## Methods

### Multiobjective Optimization Problems

An MOP can be formulated as


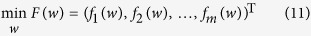


which subjects to *w* = (*w*_1_, *w*_2_, …, *w*_*n*_) ∈ Γ, where *w* is called the decision vector, and Γ is the feasible region in the decision space. In general, the objective in an MOP conflict with one another, which means a single solution does not exist in feasible space when minimize all the objectives simultaneously. Thus, for an MOP, its aim is to find Pareto optimal solutions.

Without loss of generality, we consider a minimization problem. Given two points *w*_*a*_, *w*_*b*_ ∈ Γ, *w*_*a*_ dominates *w*_*b*_ (witten as 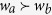), iff *f*_*i*_(*w*_*a*_) ≤ *f*_*i*_(*w*_*b*_) for all *i* = 1, 2, …, *m*, and *f*_*j*_(*w*_*a*_) < *f*_*j*_(*w*_*b*_) for at least one *j* = 1, 2, …, *m*. The set of all Pareto optimal solutions is called Pareto optimal set which defined as follows:


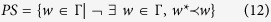


where w* is a Pareto optimal solution to equation (11) if there does not exist another solution *w* in Γ that dominates w*. The PS in the objective space is called the PF which is defined as


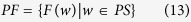


### Multiobjective Evolutionary Algorithm for Network Reconstruction

Since EAs^31^,^32^,^33^ are the most popular optimization method for handling MOPs, here, we design a multiobjective EA to solve the above MNRP, termed as MOEANet. Although we do not specify the MOEA and any state-of-the-art MOEA can be used, such as multiobjective evolutionary algorithm based on decomposition (MOEA/D)^33^ or non-dominated sorting genetic algorithm (NSGA-II)^32^, we use soft-thresholding evolutionary multiobjective algorithm (StEMO)^31^ in our framework. Particularly, we develop a new initialization scheme.

Each candidate solution in EAs is named as a chromosome. For node *i*, the chromosome is a one dimensional vector with *N* elements, namely, (*x*_1*i*_, *x*_2*i*_, …, *x*_*Ni*_). *t* is the current generation number and *P*^*t*^ is the population at the *t*th generation. EAs can return a set of solutions on the PF. Each point on the PF represents a certain local network structure. To find the best solution to decision makers, we employ an angle-based method^31^,^36^ to locate knee regions on the PF. Knee regions are solutions that have the maximum marginal rates of return, i.e., for which an improvement in one objective causes a severe degradation in another. Because MOPs always involve contradictory objective functions, decision makers often prefer their solutions to lie in these knee areas. The procedure of MOEANet is shown in Algorithm 1.


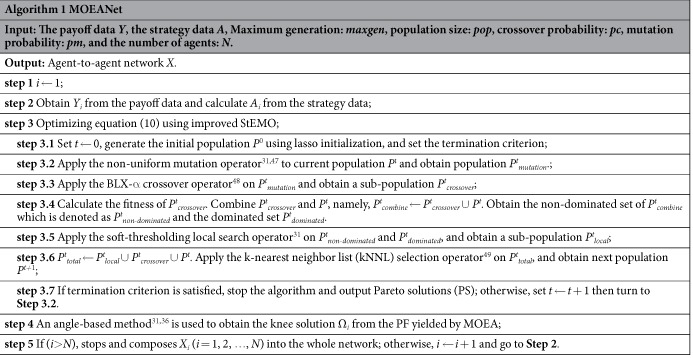


### Initialization Operator for MOEA

In this paper, we design a new initialization operator to initialize population. Consider the *L*_1_-minimization algorithm lasso^2^,^50^, which solves equation (9). Clearly, different choices for λ in equation (9) will yield different optimal solutions, so, we can obtain a set of solutions by using the lasso with different values of λ. For example, to generate *pop* individuals, we need to set *pop* different value of λ_*i*_ ∈ [0, 1], *i* = 1, 2, 3, …, *pop*, and equation (9) is then solved by the lasso. This procedure is described in Algorithm 2.


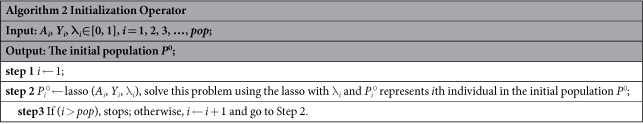


### Solution Selection

EAs can return a set of solutions on the PF. Each point on the PF represents a certain local network structure. To find the best solution to decision makers, we employ a selection strategy based on knee regions. Knee regions are solutions that have the maximum marginal rates of return, i.e., for which an improvement in one objective causes a severe degradation in another. An angle-based method^31^,^36^, for locating the knee regions on the PF, is considered in this paper. The procedure of this method is summarized as follows.

First, we normalize the PF by its maximum measurement error and ||*X*_*i*_||_1_ value. Then, we perform smoothing by interpolating the PF using B-splines and then evenly resampling from the smooth spline^31^. Finally, the knee regions can be found from this interpolated curve PF*. After finding knee regions on the PF*, we can estimate the knee areas on the original PF by finding the point on the PF that is the closest to the knee point of the PF*.

**Figure 5:**
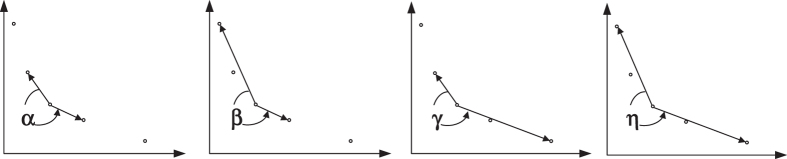
Four angles in the angle-based method.

The angle of a solution is determined by its four neighborhood solutions, as shown in Fig. 5. First four angles, α, β, γ, and η are computed, and then the largest angle among the four angles is assigned to the solution^36^. The knee point is selected by comparing the angles of solutions along the Pareto front to find the solution with the largest angle.

## Additional Information

**How to cite this article**: Wu, K. *et al*. Reconstructing Networks from Profit Sequences in Evolutionary Games via a Multiobjective Optimization Approach with Lasso Initialization. *Sci. Rep.* **6**, 37771; doi: 10.1038/srep37771 (2016).

**Publisher's note:** Springer Nature remains neutral with regard to jurisdictional claims in published maps and institutional affiliations.

## Supplementary Material

Supplementary Materials

## Figures and Tables

**Table 1:** Performance of MOEANet on eight real networks.

Name	TPR	FPR	Precision	RE	AUROC	AUPR
football	0.928 ± 0.010	0.000 ± 0.001	0.997 ± 0.004	0.067 ± 0.008	0.987 ± 0.001	0.980 ± 0.002
netscience	0.986 ± 0.003	0.000 ± 0.000	1.000 ± 0.000	0.000 ± 0.001	0.993 ± 0.002	0.986 ± 0.002
polbooks	0.978 ± 0.005	0.000 ± 0.000	1.000 ± 0.000	0.009 ± 0.001	0.989 ± 0.002	0.983 ± 0.004
dolphin	0.972 ± 0.004	0.000 ± 0.000	1.000 ± 0.000	0.013 ± 0.002	0.986 ± 0.002	0.979 ± 0.003
ZK	0.974 ± 0.002	0.000 ± 0.000	1.000 ± 0.000	0.013 ± 0.001	0.964 ± 0.005	0.948 ± 0.007
lesmis	0.981 ± 0.002	0.000 ± 0.000	1.000 ± 0.000	0.013 ± 0.002	0.990 ± 0.001	0.985 ± 0.002
adjnoun	0.975 ± 0.007	0.000 ± 0.000	1.000 ± 0.000	0.011 ± 0.002	0.987 ± 0.004	0.980 ± 0.006
neuralnet	0.955 ± 0.003	0.000 ± 0.000	0.988 ± 0.003	0.008 ± 0.000	0.977 ± 0.001	0.948 ± 0.000

*N*_*M*_ is set to 1.6 for all of real networks.

The performance of MOEANet is measured in terms of TPR, FPR, Precision, RE, AUROC, and AUPR (see Supplementary Note 1). Each solution of sub-problem is selected from the PF based on knee regions. Each result is obtained by averaging over 30 independent realizations. More details of the real networks can be found in Supplementary Table S1. The parameters of MOEANet are showed in Supplementary Table S2.
