# Supplementary material for: Reconstructing Networks from Profit Sequences in Evolutionary Games via a Multiobjective Optimization Approach with Lasso Initialization
Source: Sci Rep. 2016 Nov 25;6:37771. doi: 10.1038/srep37771 (PMC5122890; doi:10.1038/srep37771)
Supplement: Supplementary Materials [file srep37771-s1.pdf]

Supplementary Materials for Reconstructing Networks from Profit Sequences in Evolutionary Games via a Multiobjective Optimization Approach with Lasso Initialization

Kai Wu[1], Jing Liu[1], and Shuai Wang[1]

Key Laboratory of Intelligent Perception and Image Understanding of Ministry of Education, Xidian University, Xi'an 710071, China. Correspondence and requests for materials should be addressed to Jing Liu (email: neouma@163.com)

Contents

1. **Supplementary Note 1: Performance Measures**
2. **Supplementary Note 1: Numerical Simulation of EG**
3. **Supplementary Figures**
4. **Supplementary Table**
5. **Supplementary References**

Supplementary Note 1: Performance Measures

To quantify the performance of our reconstruction method, two standard measurement indices are introduced, namely, the area under the receiver operating characteristic curve (AUROC) and the area under the precision-recall curve (AUPR) [S1].

True positive rate (TPR), false positive rate (FPR), Precision and Recall used to calculate AUROC and AUPR are defined as follows:

$$\mathrm{TPR}(k) = \frac{\mathrm{TP}(k)}{\mathrm{G}} = \mathrm{Recall}(k) \tag{S1}$$

where k is the cutoff in the edge list, TP(k) is the number of true positives in the top k predictions in the edge list, and G is the number of positives in the gold standard.

$$\mathrm{FPR}(k) = \frac{\mathrm{FP}(k)}{\mathrm{Q}} \tag{S2}$$

where FP(k) is the number of false positive in the top k predictions in the edge list, and Q is the number of negatives in the gold standard.

$$\mathrm{Precision}(k) = \frac{\mathrm{TP}(k)}{\mathrm{TP}(k) + \mathrm{FP}(k)} \tag{S3}$$

Reconstruction error (RE) is used to compare the weight matrix of reconstructed network and target network directly,

$$\mathrm{RE} = \frac{1}{N^2} \sum_{i=1}^{N} \sum_{j=1}^{N} \left| x_{ij} - x_{ij}^{*} \right| \tag{S4}$$

where x_{ij} is the edge weight between nodes i and j in the target network, x_{ij}^{*} is the edge weight between nodes i and j in reconstructed networks, and N is network size.

Supplementary Note 2: Numerical Simulation of EG

Numerical simulation of EG is described as follows. Initially, a fraction of agents is set to choose the strategy of cooperation and the remaining agents are set to choose the strategy of defection. Nodal states are updated in parallel. For agent i of degree $\langle k \rangle$, at round t, the payoff of this agent is calculated. To maximize the payoff of agent i, its strategy is updated. A Monte Carlo round t is referred to the situation where all the states at t+1 have been updated according to their states at t. Random link weights are chosen uniformly from the interval [1.0, 6.0].

Supplementary Figures

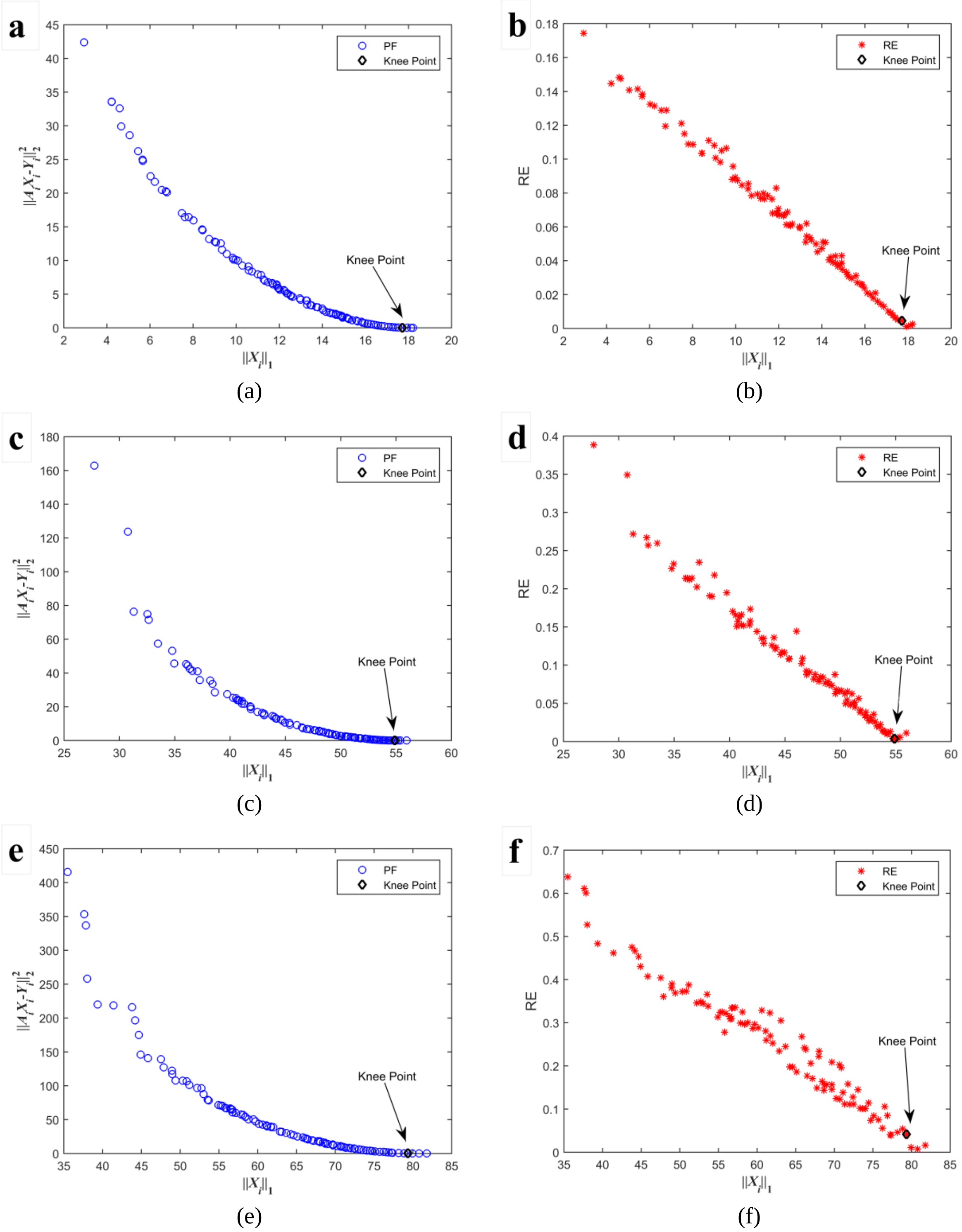

Supplementary Figure S1. The relationships between measurement error, RE, and the sparsity of the solutions on the PF and the position of knee point for three different $\langle k \rangle$. (a) (b) $\langle k \rangle$=6. (c) (d) $\langle k \rangle$=12. (e) (f) $\langle k \rangle$=18. Supplementary Figure S1 plots the relationship between measurement error and $||X_i||_1$ when the average degree of ER networks changes. The simulations are conducted on ER networks with 100 nodes. Here, the degree of networks $\langle k \rangle$ is increased from 6 to 18 in steps of 6, N_M=1.4, and σ=0.05. In each case,

the left-hand graph is a 2-D plot, graphing the relationship between the measurement error and $||X_i||_1$. The right-hand graph shows one 2-D views of the data; variation of RE with change in sparsity $||X_i||_1$. Each graph of Supplementary Fig. S1 shows results for one example trial.

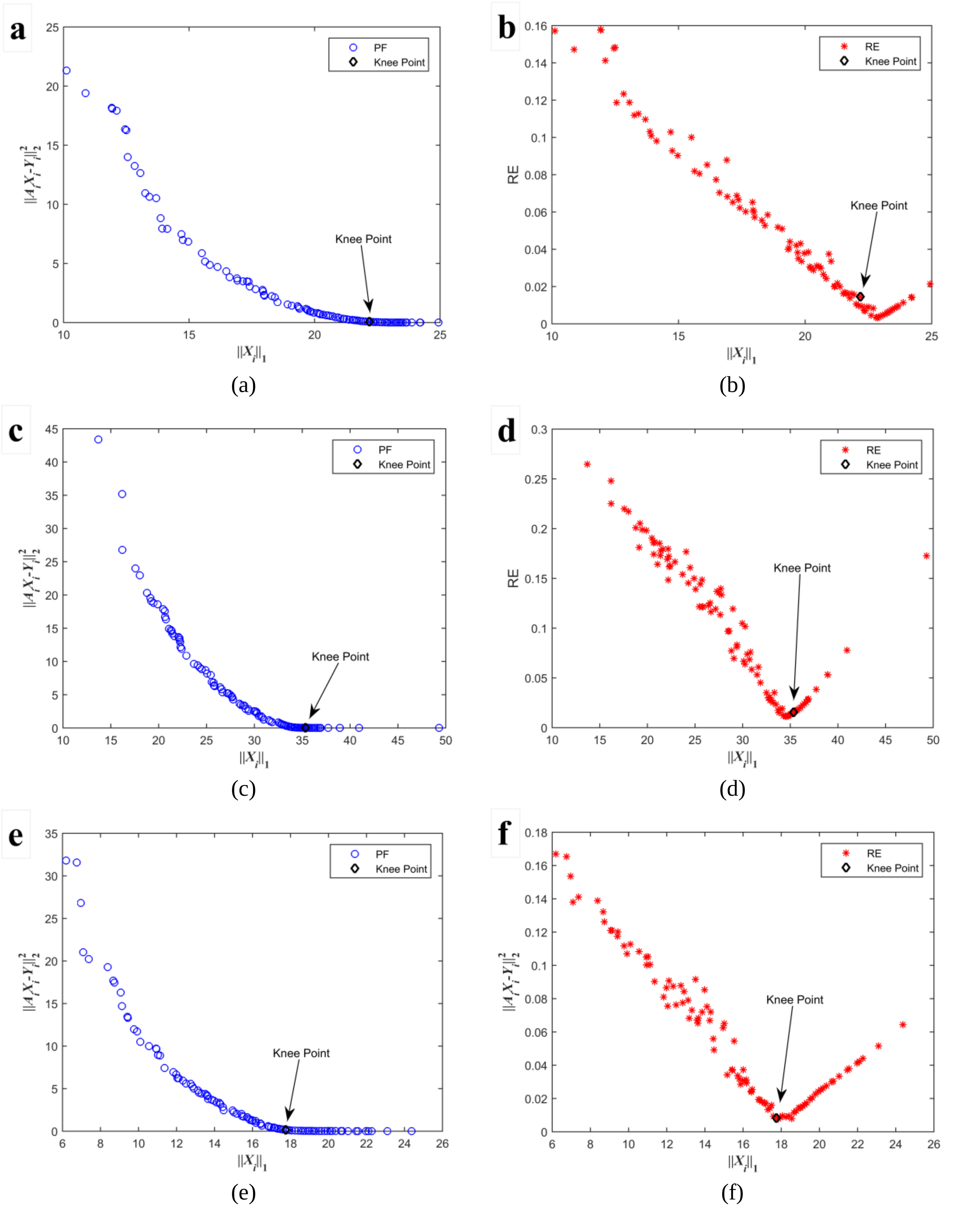

Supplementary Figure S2. The relationships between measurement error, *RE*, and the sparsity of the solutions on the PF and the position of knee point for three different strength of noise. Different variance σ^2 of Gaussian white noise $N(0, \sigma^2)$ are embedded in time series for obtaining vector Y_i. (a) (b) σ=0.1. (c) (d) σ=0.2. (e) (f) σ=0.3. The simulations are conducted on ER networks with 100 nodes, $\langle k \rangle$=12, and N_M=1.2. We have gathered data for three different cases where σ varies from 0.1 to 0.3 in increments of size 0.1.

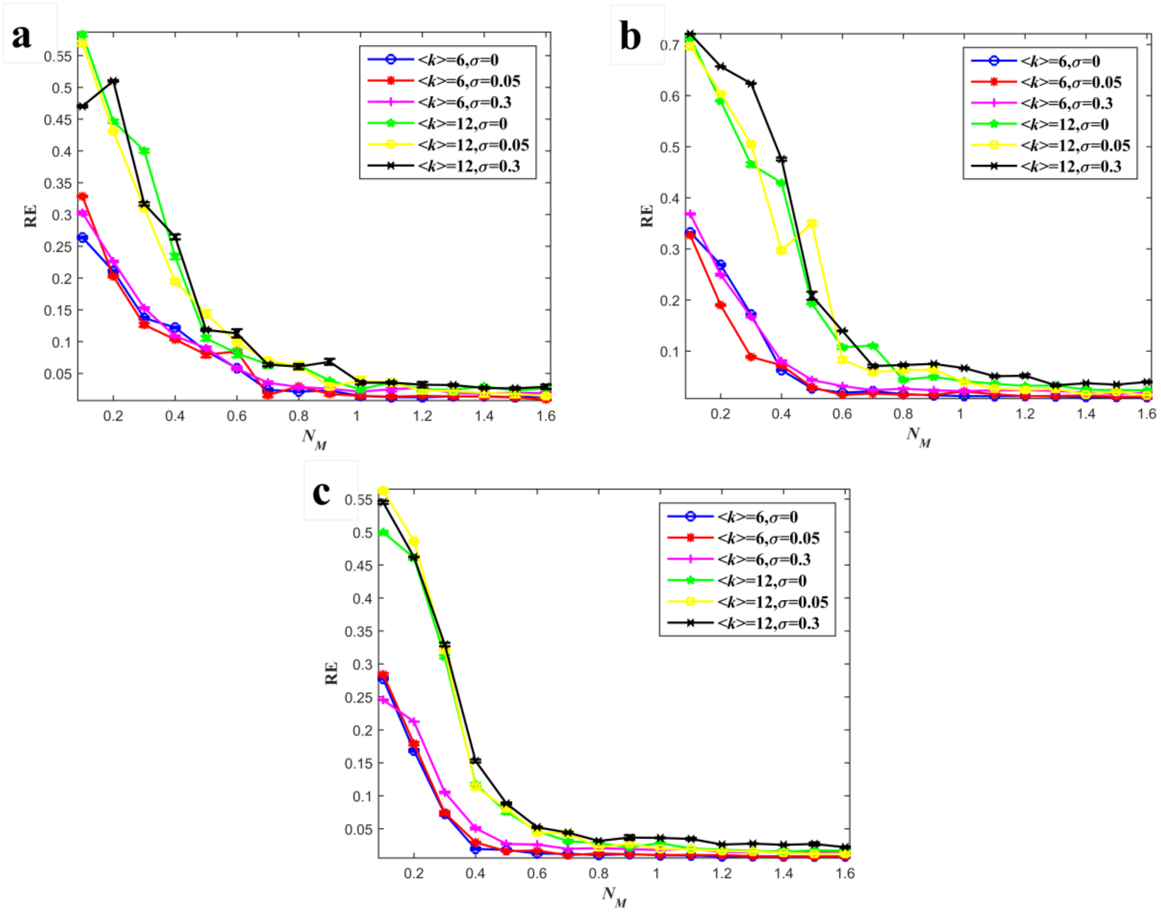

Supplementary Figure S3. RE as a function of the relative data length N_M of time series for (a) BA networks, (b) NW networks, and (c) WS networks. We simulate evolutionary games on different model-based networks, including weighted Barab ási-Albert scale-free networks (BA) [S2], weighted Newman-Watts small-world networks (NW) [S3], and weighted Watts-Strogatz small-world networks (WS) [S4]. The simulations are conducted on network size N=100, $\langle k \rangle$=6 and 12, and σ=0, 0.05, and 0.3. Here, N_M is increased from 0.1 to 1.6 in steps of 0.1. Rewriting probability of small-world networks is 0.3. Each data point is obtained by averaging over 30 independent realizations. each solution of sub-problem is selected from the PF based on knee regions.

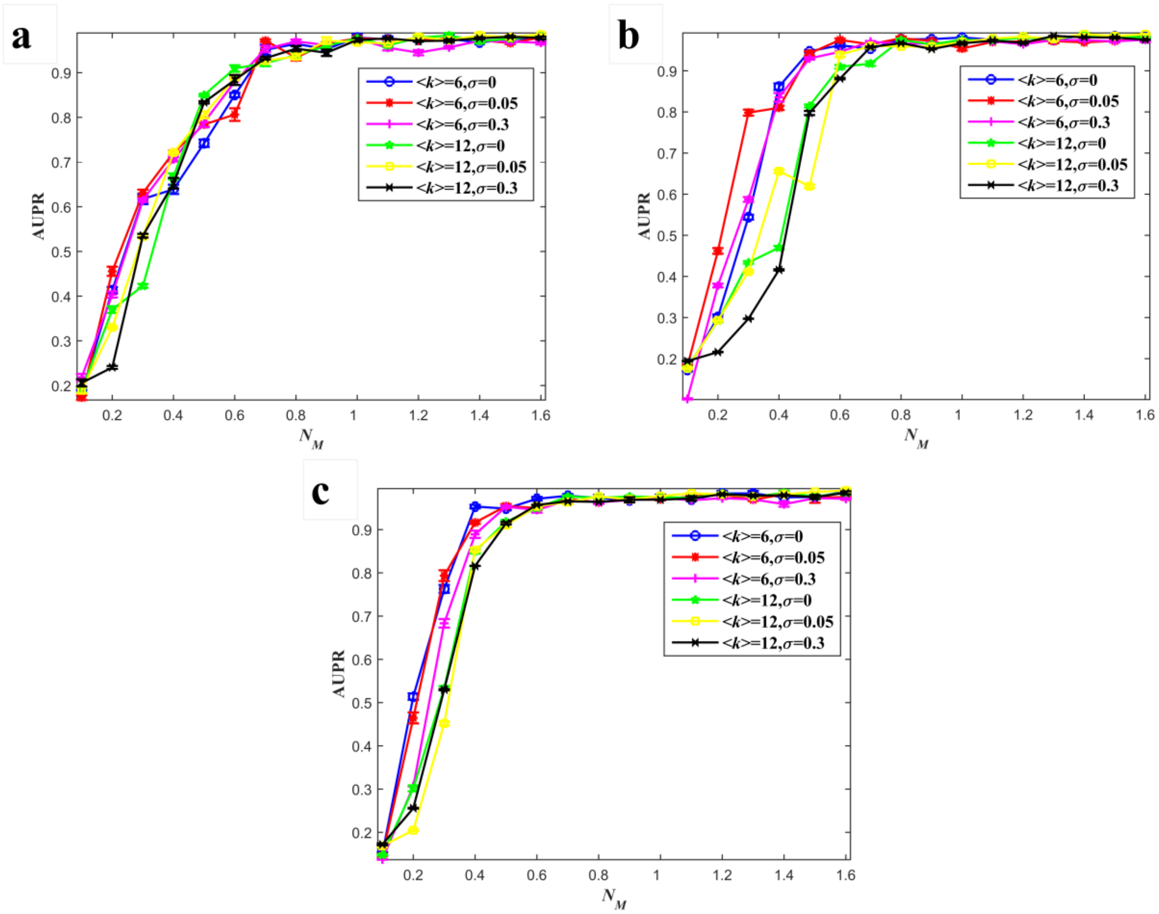

Supplementary Figure S4. AUPR as a function of the relative data length N_M of time series for (a) BA networks, (b) NW networks, and (c) WS networks. We simulate evolutionary games on different model-based networks, including weighted BA networks, weighted NW small-world networks, and weighted WS small-world networks. The simulations are conducted on network size N=100, $\langle k\rangle$=6 and 12, and σ=0, 0.05, and 0.3. Here, N_M is increased from 0.1 to 1.6 in steps of 0.1. Rewriting probability of small-world networks is 0.3. Each data point is obtained by averaging over 30 independent realizations. each solution of sub-problem is selected from the PF based on knee regions.

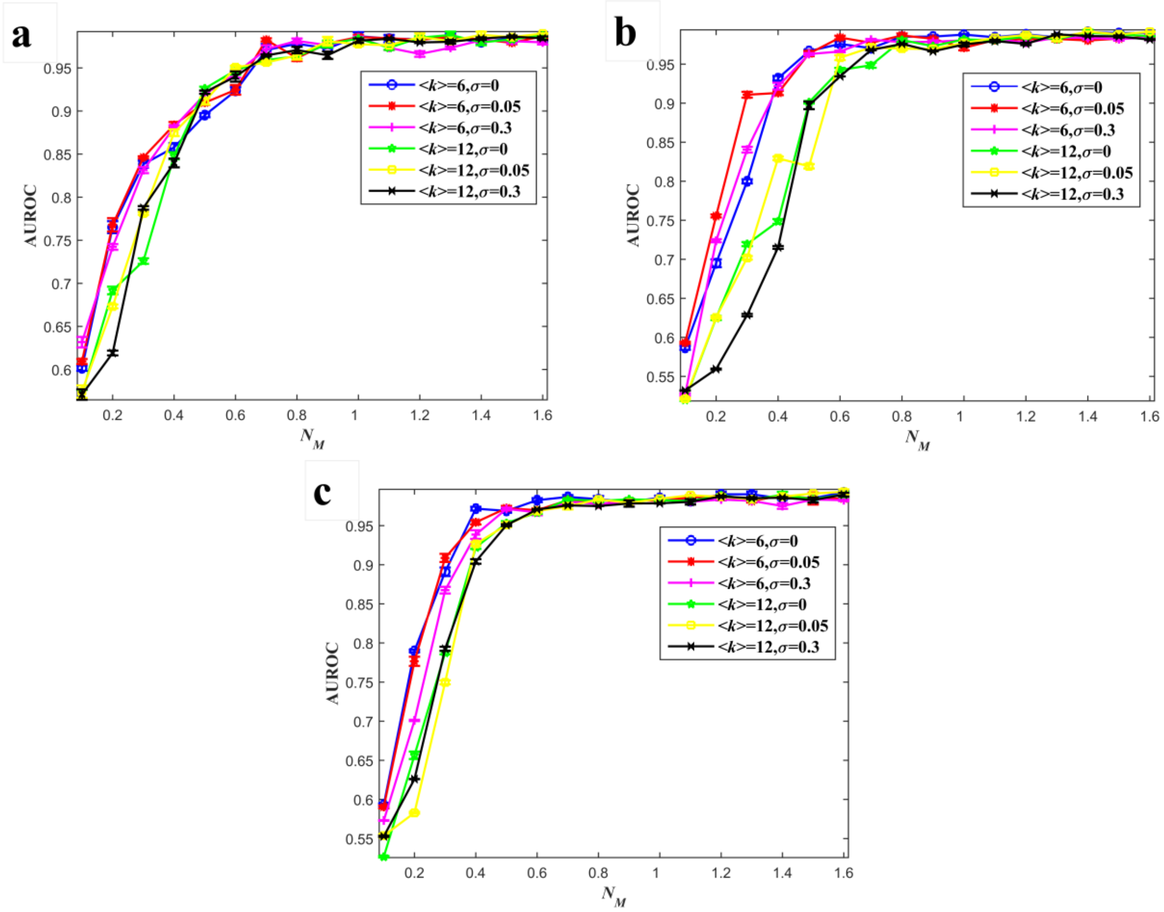

Supplementary Figure S5. AUROC as a function of the relative data length N_M of time series for (a) BA networks, (b) NW networks, and (c) WS networks. We simulate evolutionary games on different model-based networks, including weighted BA networks, weighted NW small-world networks, and weighted WS small-world networks. The simulations are conducted on network size N=100, $\langle k \rangle$=6 and 12, and σ=0, 0.05, and 0.3. Here, N_M is increased from 0.1 to 1.6 in steps of 0.1. Rewriting probability of small-world networks is 0.3. Each data point is obtained by averaging over 30 independent realizations. each solution of sub-problem is selected from the PF based on knee regions.

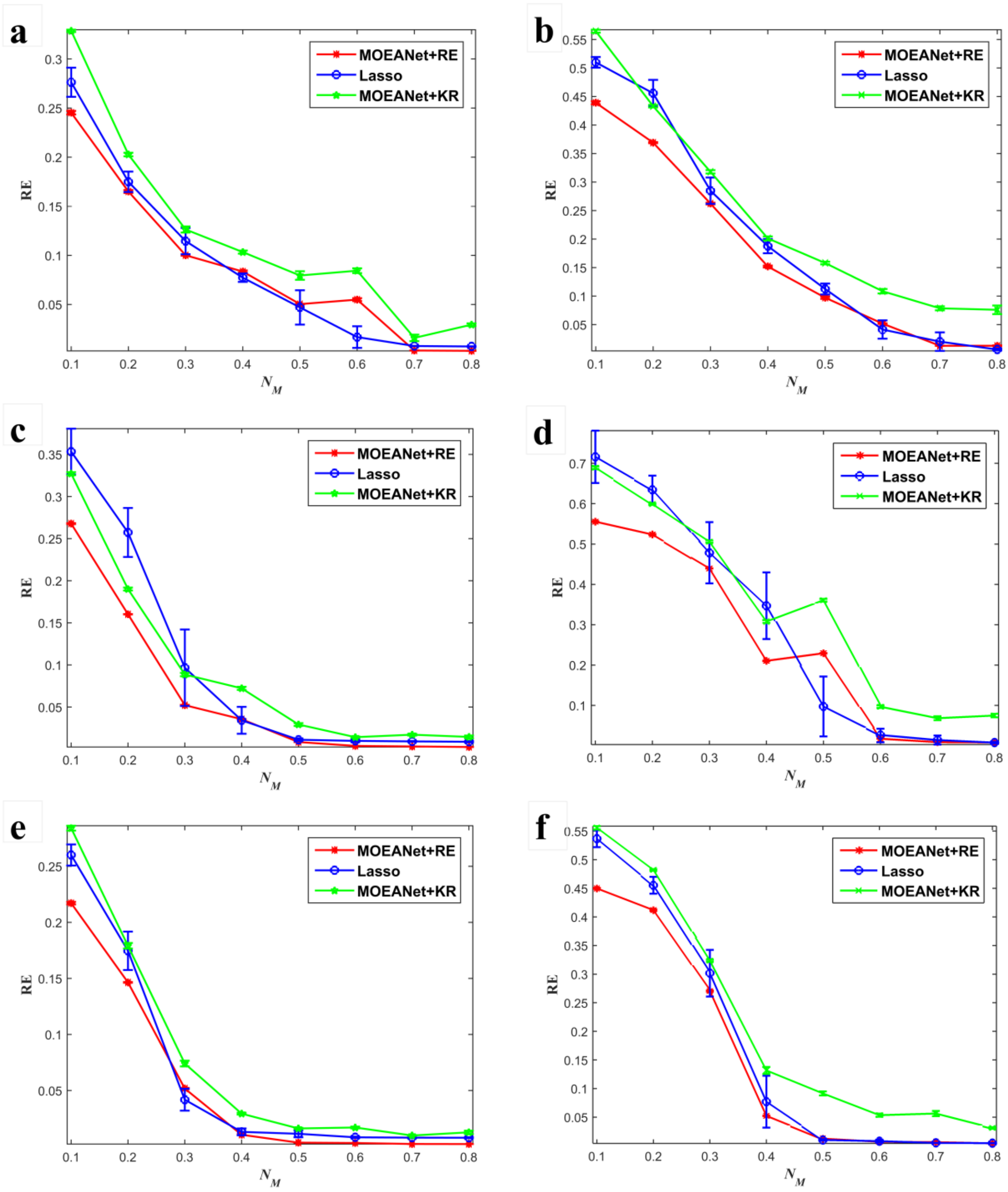

Supplementary Figure S6. RE as a function of the relative data length N_M of time series for (a) BA networks with $\langle k\rangle$=6, (b) BA networks with $\langle k\rangle$=12, (c) NW networks with $\langle k\rangle$=6, (d) NW networks with $\langle k\rangle$=12, (e) WS networks with $\langle k\rangle$=6, and (f) WS networks with $\langle k\rangle$=12. Rewriting probability of small-world networks is 0.3. Here, N=100, σ=0.05. N_M is increased from 0.1 to 0.8 in steps of 0.1. For MOEANet+RE, each solution of sub-problem selected from the PF has the best *generalization ability*, namely, the smallest value of *RE*. For MOEANet+KR, each solution of sub-problem is selected from the PF based on knee regions. For the lasso , we set λ=0.001 for all reconstructions. Each data point is obtained by averaging over 30 independent realizations.

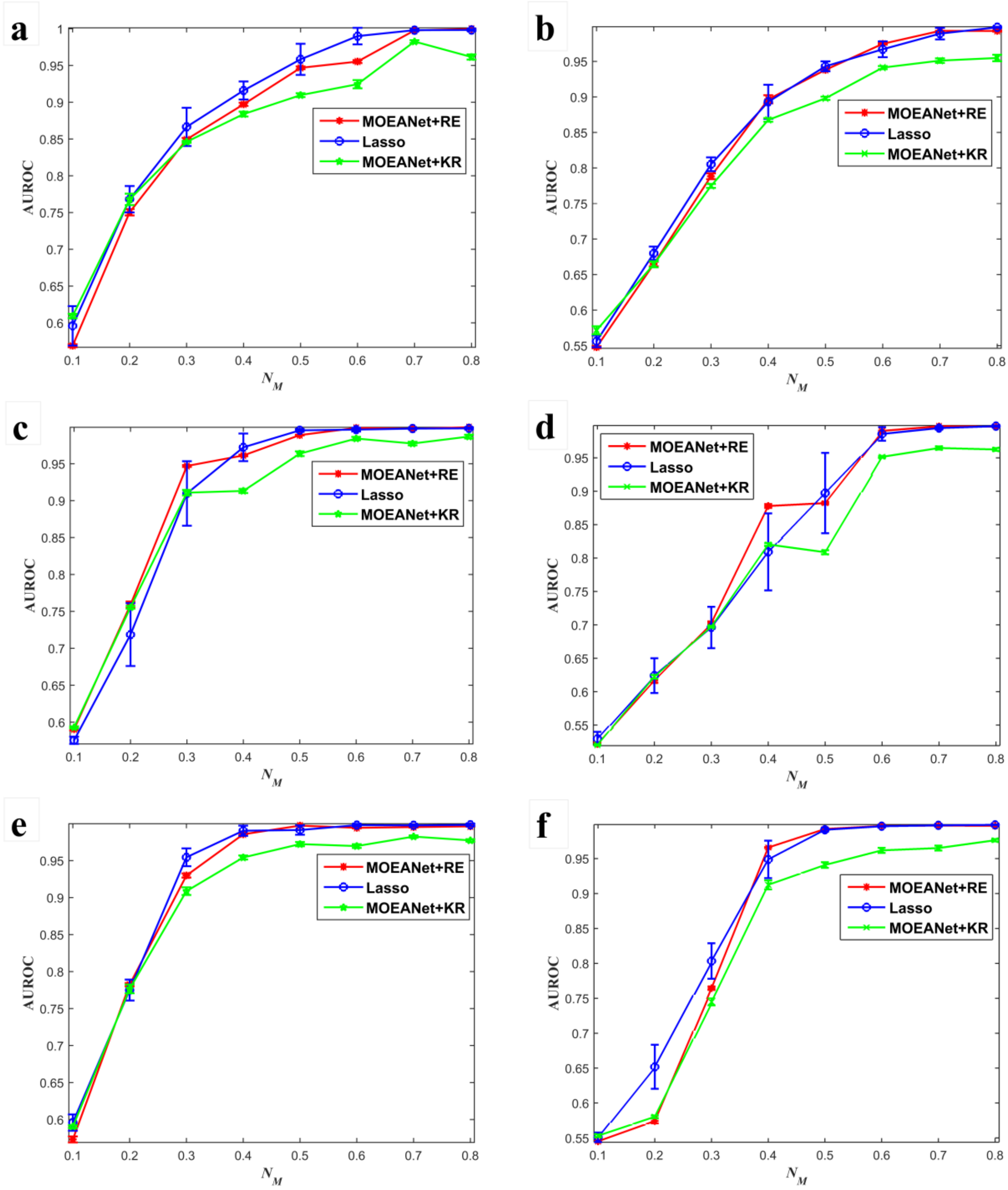

Supplementary Figure S7. AUROC as a function of the relative data length N_M of time series for (a) BA networks with $\langle k \rangle$=6, (b) BA networks with $\langle k \rangle$=12, (c) NW networks with $\langle k \rangle$=6, (d) NW networks with $\langle k \rangle$=12, (e) WS networks with $\langle k \rangle$=6, and (f) WS networks with $\langle k \rangle$=12. Rewriting probability of small-world networks is 0.3. Here, N=100, σ=0.05. N_M is increased from 0.1 to 0.8 in steps of 0.1. For MOEANet+RE, each solution of sub-problem selected from the PF has the best *generalization ability*, namely, the smallest value of *RE*. For MOEANet+KR, each solution of sub-problem is selected from the PF based on knee regions. For the lasso , we set λ=0.001 for all reconstructions. Each data point is obtained by averaging over 30 independent realizations.

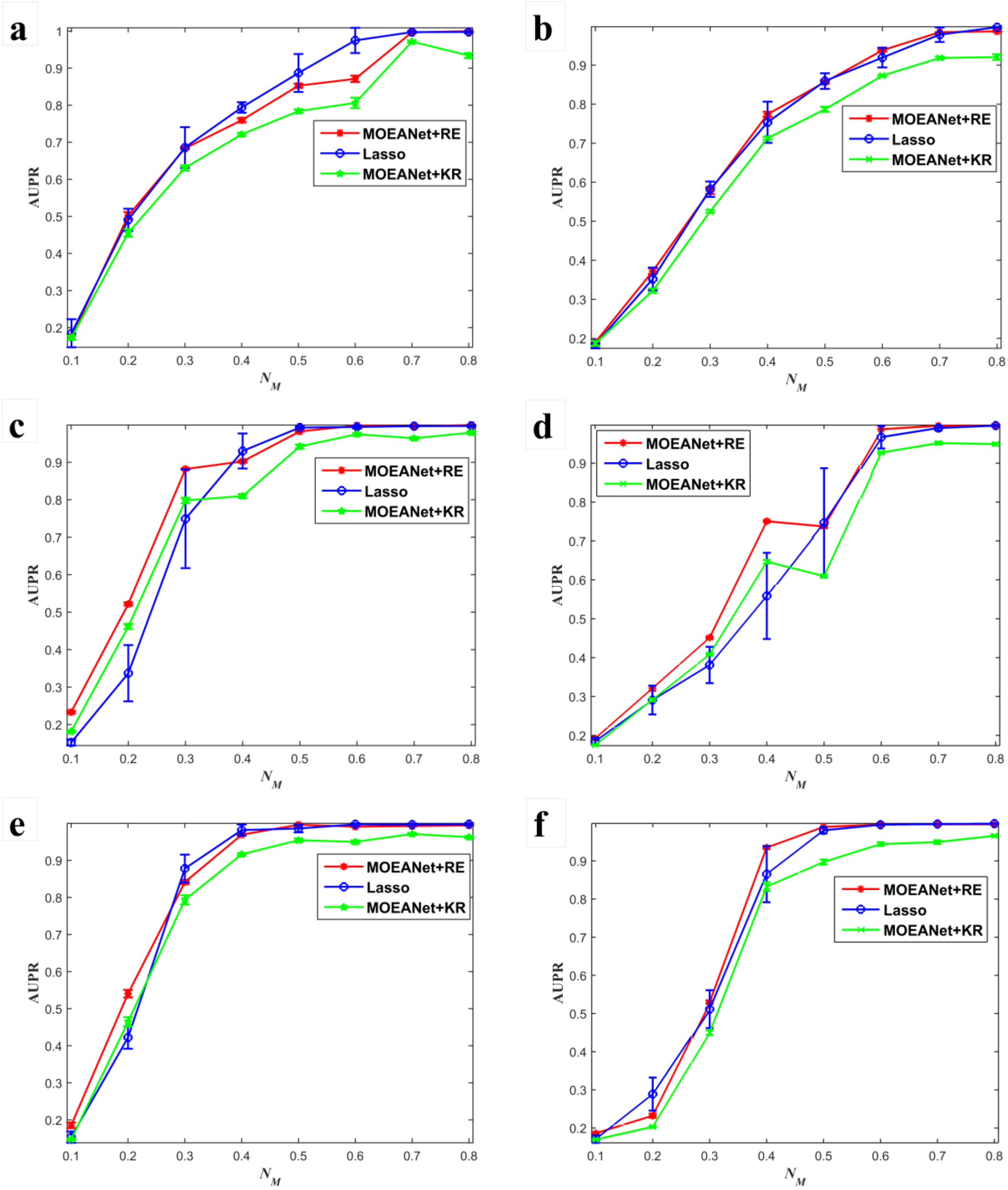

Supplementary Figure S8. AUPR as a function of the relative data length N_M of time series for (a) BA networks with $\langle k \rangle$=6, (b) BA networks with $\langle k \rangle$=12, (c) NW networks with $\langle k \rangle$=6, (d) NW networks with $\langle k \rangle$=12, (e) WS networks with $\langle k \rangle$=6, and (f) WS networks with $\langle k \rangle$=12. Rewriting probability of small-world networks is 0.3. Here, N=100, σ=0.05. N_M is increased from 0.1 to 0.8 in the step of 0.1. For MOEANet+RE, each solution of sub-problem selected from the PF has the best *generalization ability*, namely, the smallest value of *RE*. For MOEANet+KR, each solution of sub-problem is selected from the PF based on knee regions. For the lasso , we set λ=0.001 for all reconstructions. Each data point is obtained by averaging over 30 independent realizations.

Supplementary Table

The details of the real social networks studied in this paper are presented in Supplementary Table S1, which includes the number N of nodes, the number L of links, and description of the networks.

Supplementary Table S1. Description of the real social networks analyzed in the paper.

Name	N	L	Description
football [S5]	115	613	The network of American football games, Fall 2000.
netscience[S6]	1589	2742	A coauthorship network of scientists working on networks.
polbooks [S7]	105	441	A network of books about US politics.
dolphin [S8]	62	159	Social network of dolphins.
ZK [S9]	34	78	Social network of friendships of a karate club.
lesmis [S10]	77	254	Coappearance network of characters in the novel *Les Miserables*.
adjnoun [S6]	112	425	Network of common adjectives and nouns in the novel *David Copperfield*
neuralnet [S4]	297	2359	Represent the neural network of C. Elegans. Data.

The parameters of MOEANet are showed in **Supplementary Table S2**.

Supplementary Table S2. Parameter settings of MOEANet.

Parameter	Meaning	Value
maxgen	The maximum generation	150
pop	Population size	100
pm	Mutation rate	0.1
pc	Crossover rate	0.5

Supplementary References

[S1] Grau, J., Grosse, I. & Keilwagen, J. PRROC: computing and visualizing precision-recall and receiver operating characteristic curves in R. *Bioinformatics* **31**, 2595-2597 (2015).

[S2] Barabási, A. L. & Albert, R. Emergence of scaling in random networks. *Science* **286**, 509-512 (1999).

[S3] Newman, M. E. & Watts, D. J. Renormalization group analysis of the small-world network model. *Physics Letters A* **263**, 341-346 (1999).

[S4] Watts, D. J. and Strogatz, S. H. Collective dynamics of ‘small-world’ networks. *Nature* **393**, 440-442 (1998).

[S5] Girvan, M. and Newman, M. E. Community structure in social and biological networks. *Proc. Natl. Acad. Sci.* **99**, 7821-7826 (2002).

[S6] Newman, M. E. Finding community structure in networks using the eigenvectors of matrices. *Phys. Rev. E* **74**, 036104 (2006).

[S7] V. Krebs, http://www.orgnet.com/divided.html.

[S8] Lusseau, D. *et al.* The bottlenose dolphin community of doubtful sound features a large proportion of long-lasting associations. *Behav. Ecol. Sociobiol.* **54**, 396-405 (2003).

[S9] Zachary, W. W. An information flow model for conflict and fission in small groups. *J. Anthropol. Res.* 452-473 (1977).

[S10] Knuth, D. E. *The Stanford Graph Base: A Platform for Combinatorial Computing*. (Addison-Wesley, Reading, MA, 1993).
